# Circulating Interleukin-36 Alpha, Beta, Gamma, and Interleukin-37 Levels in Patients With Chronic Coronary Syndrome

**DOI:** 10.14740/cr2285

**Published:** 2026-10-24

**Authors:** Dinaldo Cavalcanti de Oliveira, Sabrina Barboza, Carolina Oliveira, Ernesto Osterne, Thomas Osterne, Evandro Osterne, Paulo Motta

**Affiliations:** a Internal Medicine Department, Hospital das Clinicas, Federal University of Pernambuco, Recife-PE, 50670-901, Brazil

**Keywords:** Interleukins 36 alpha, Interleukin 36 beta, Interleukin36 gamma, Interleukin 37, Chronic coronary syndrome

## Abstract

**Background:**

The aim of this study was to assess whether circulating interleukin (IL)-36α, IL-36β, IL-36γ, and IL-37 levels were higher in patients with chronic coronary syndrome compared with healthy controls.

**Methods:**

This was a cross-sectional, descriptive, and analytical study, with recruitment conducted from August 2022 to February 2023. After undergoing coronary angiography, patients were assessed in the hemodynamics laboratory according to the inclusion and exclusion criteria. For those who met these criteria, the study was explained, and they were invited to participate. Clinical characteristics were collected via interview, followed by a blood sample for the measurement of ILs. Serum IL levels were quantified using a sandwich ELISA assay (R&D Systems, USA), according to the manufacturer’s recommendations. The present study was submitted to and approved by the institutional research ethics committee.

**Results:**

A total of 100 patients were recruited (mean age: 58 ± 6.2 years; 59% male, 41% female) along with 100 healthy controls (mean age: 56 ± 4.6 years, 59% male, 41% female). Comparative analyses of circulating IL-36 family levels between patients and healthy controls revealed the following: IL-36α, 6.25 pg/mL (2.25–81.68) vs. 6.25 pg/mL (6.25–127.6) (P = 0.6); IL-36β, 9.68 pg/mL (6.25–36.39) vs. 6.25 pg/mL (6.25–6.25) (P = 0.006); and IL-36γ, 3,212 pg/mL (2,091–5,761) vs. 89.1 pg/mL (30.98–145.29) (P < 0.001), respectively.

**Conclusions:**

Circulating IL-36α and IL-36β levels were higher in patients with chronic coronary syndrome compared with healthy controls. These ILs may be involved in the development of coronary atherosclerosis. However, further studies are needed to clarify the role of these ILs.

## Introduction

Coronary artery disease (CAD) is a major global public health concern and one of the leading causes of cardiovascular mortality worldwide. The prevalence of CAD among individuals older than 20 years is 7.1% and is higher in men than in women across all age groups [1].

Diseases of the circulatory system place a considerable burden on health systems and government budgets. In 2022, 1.68 million deaths in the European Union were attributed to circulatory system diseases, accounting for 32.7% of all deaths. This percentage was considerably higher than that attributed to cancer, which represented the second most common cause of death (malignant neoplasms; 22.5% of all deaths) [2].

Coronary atherosclerosis is a chronic, low-grade inflammatory disease characterized by atheroma formation in the arterial intima. Genetic conditions and risk factors lead to endothelial dysfunction associated with an immune-inflammatory response, resulting in the deposition of cholesterol-containing lipoproteins; leukocyte recruitment; activation of adaptive and innate immunity; release of pro- and anti-inflammatory mediators, apoptotic substances, pro- and anti-inflammatory interleukins (ILs), and vascular angiogenesis factors; mineralization; extracellular matrix formation; migration and activation of smooth muscle cells; and other events [3].

CAD may lead to clinical events through the progressive development of stenoses that narrow the arterial lumen and/or by the sudden formation of an occlusive or subocclusive thrombus on the surface of the atheromatous plaque, particularly in plaques with a high inflammatory burden [4].

The IL-36 family has been described as having proinflammatory activity in diseases such as systemic lupus erythematosus, rheumatoid arthritis, psoriasis, inflammatory bowel disease, Crohn’s disease, chronic obstructive pulmonary disease, and others [5–8].

Because the inflammatory activity of IL-36 has been shown to contribute to the pathophysiology of several systemic inflammatory diseases of varying severity, this family of ILs may also be involved in other systemic inflammatory diseases beyond those already described [9].

Based on this premise, we recruited patients with CAD to evaluate whether circulating levels of ILs in the IL-36 family and IL-37 were higher in these patients compared with healthy controls.

## Materials and Methods

This was a cross-sectional, descriptive, and analytical study, with recruitment conducted from August 2022 to February 2023.

Patients with chronic coronary syndrome and healthy controls matched for sex and age (± 5 years) were recruited. The sample size was determined empirically.

The inclusion criteria were as follows: age > 18 years, clinical diagnosis of chronic coronary syndrome under optimized medical therapy, clinical indication for coronary angiography, and the presence of at least one coronary artery stenosis ≥ 50% on angiography. Patients were required to meet all inclusion criteria to be included in the study.

Patients were excluded if they met at least one of the following criteria: prior surgical or percutaneous myocardial revascularization, severe liver disease, stage IV or V chronic kidney disease, blood dyscrasia, active cancer, active metastasis, history of chemotherapy or radiotherapy, life expectancy < 1 year, current use of immunosuppressive therapy, psoriasis, inflammatory bowel disease, rheumatoid arthritis, or systemic lupus erythematosus.

Standard materials were used for biological sample (blood) collection, including a plain tube containing separator gel for blood collection, one pair of procedure gloves, one 21- or 23-gauge butterfly needle or one 30 × 6 or 30 × 7 needle, one 10-mL syringe, one tourniquet, two cotton balls, and 5 mL of 70% alcohol. Plain tubes containing separator gel were used to obtain serum.

The vein to be punctured for sample collection was identified by visual inspection for a large-caliber vein; by instructing the participant to lower the arm and open and close the hand; by gently massaging the upper limb from the wrist toward the elbow; by palpating the vein with the index finger, avoiding palpation with the thumb; or by stabilizing the vein with the fingers in cases of flaccidity. Tapping the patient’s vein with the fingers to facilitate vein localization is contraindicated, as it may cause hemolysis and thereby compromise the integrity of the blood sample.

Serum ILs levels were quantified using a sandwich ELISA assay (R&D Systems, USA), according to the manufacturer’s recommendations.

After undergoing coronary angiography, patients were assessed in the hemodynamics laboratory according to the inclusion and exclusion criteria. For those who met these criteria, the study was explained, and they were invited to participate. Patients who agreed to participate subsequently signed the informed consent form.

The clinical characteristics of the patients were collected using a structured questionnaire. A blood sample was then obtained as described above and sent to the research laboratory for processing.

Categorical variables were presented as absolute and relative frequencies. For numerical variables, the Shapiro–Wilk test was used to assess normality. Variables with a normal distribution were expressed as the mean and standard deviation, whereas non-normally distributed variables were expressed as the median and 25th and 75th percentiles.

Continuous variables were compared using Student’s *t*-test when normally distributed and the Mann–Whitney test when non-normally distributed. A P value ≤ 0.05 was considered statistically significant.

The present study was submitted to and approved by the Institutional Research Ethics Committee. The study was conducted in accordance with the Declaration of Helsinki for clinical research and with the national regulations governing clinical research in the country in which it was performed.

## Results

A total of 100 patients were recruited (mean age: 58 ± 6.2 years; 59% male, 41% female). One hundred healthy controls were also included, with a mean age of 56 ± 4.6 years (59% male, 41% female).

The main clinical and demographic characteristics were as follows: hypertension in 79 patients (79%), diabetes mellitus in 41 (41%), dyslipidemia in 31 (31%), previous myocardial infarction in 27 (27%), and previous stroke in 22 (22%). In addition, 84 patients (84%) were married, 38 (38%) were current smokers, 64 (64%) had a monthly income of ≤ 1 minimum wage, and 14 (14%) had completed higher education (Tables 1, 2).

**Table 1:** Clinical Characteristics of the Patients

Variable	Number of patients, (%)
Hypertension	79 (79%)
Diabetes mellitus	41 (41%)
Dyslipidemia	31 (31%)
Previous AMI	27 (27%)
Previous CVA	22 (22%)
Previous cancer	22 (22%)
Previous CVD	12 (12%)

AMI: acute myocardial infarction, CVA: cerebrovascular accident, CVD: cardiovascular disease.

**Table 2:** Sociocultural and Socioeconomic Characteristics of the Patients

Variable	Number of patients, (%)
Married	84 (84%)
Smokers	38 (38%)
Social alcohol consumption	13 (13%)
Sedentary lifestyle	86 (86%)
Income ≤ 1 minimum wage	64 (64%)
Completed higher education	14 (14%)

No complications related to coronary angiography were documented after patient recruitment, and no complications related to study-related venipuncture for blood sample collection were observed.

Comparative analyses of circulating IL-36 family levels between patients and healthy controls revealed the following: IL-36α, 6.25 pg/mL (2.25–81.68) vs. 6.25 pg/mL (6.25–127.6) (P = 0.6); IL-36β, 9.68 pg/mL (6.25–36.39) vs. 6.25 pg/mL (6.25–6.25) (P = 0.006); and IL-36γ, 3,212 pg/mL (2,091–5,761) vs. 89.1 pg/mL (30.98–145.29) (P < 0.001), respectively.

Comparative analyses of circulating IL-36 family levels between patients with stenosis < 70% versus patients with at least one stenosis ≥ 70% revealed the following: IL-36α, 6.25 pg/mL (2.25–74.3) vs. 7.1 pg/mL (6.25–88.7) (P = 0.9); IL-36β, 7.25 pg/mL (6.25–34.9) vs. 6.25 pg/mL (6.25–37.5) (P = 0.8); and IL-36γ, 3,023 pg/mL (2,031–4,896) vs. 3,608 pg/mL (2,211–6,014) (P < 0.01), respectively.

Serum IL-37 levels did not differ significantly between patients with chronic coronary syndrome and healthy controls (15.6 pg/mL (15.6–421) vs. 15.6 pg/mL (15.6–574), respectively; P = 0.7).

## Discussion

Circulating IL-36α and IL-36β levels were higher in patients with chronic coronary syndrome compared with healthy controls.

Members of the IL-36 family, including IL-36α (IL-1F6), IL-36β (IL-1F8), and IL-36γ (IL-1F9), bind to IL-1Rrp2 and utilize IL-1RAcP as a co-receptor. IL-36α, IL-36β, IL-36γ, and IL-36Ra lack caspase cleavage sites, and N-terminal truncation at the conserved AX-Asp motif, common to IL-1 family members, markedly increases their pro-inflammatory activity [10].

IL-36 is produced by several cell types and tissues, including keratinocytes; epithelial cells of the respiratory tract, esophagus, and intestine; cardiac cells; fibroblasts; neurons; glial cells; dendritic cells; endothelial cells; monocytes; macrophages; and B and T lymphocytes [11–13].

Conversely, when low-density lipoprotein (LDL) is present in the intima of coronary arteries, it binds to proteoglycans, undergoes structural modification and oxidation, and is phagocytosed by macrophages, leading to the release of immune-inflammatory substances. This event is crucial to the pathophysiology of CAD. Activation of the inflammasome, expression of leukocyte adhesion molecules, and inflammatory ILs also contribute to atheroma formation [14].

ILs of the IL-36 family induce immune cell recruitment, antigen presentation, the production of inflammatory ILs, and mitogen-activated protein kinase (MAPK) and nuclear factor κB (NF-κB) signaling [15, 16].

IL-36 increases the levels of IL-17, IL-6, IL-1α, IL-1β, IL-12, IL-23, IL-10, granulocyte colony-stimulating factor (G-CSF), IL-18, C-X-C motif chemokine ligand 1 (CXCL1), C-C motif chemokine ligand 20 (CCL20), tumor necrosis factor (TNF), interferon gamma receptor 1 (IFNGR1), IFNGR2, IFNAR2, vascular cell adhesion molecule 1 (VCAM-1), intercellular adhesion molecule 1 (ICAM-1), IL-8, CCL2, and plasminogen activator inhibitor 1 (PAI-1), as demonstrated in studies of some non-atherosclerotic systemic inflammatory diseases [9, 11, 17].

The IL-36 family is thought to play an important role in inflammatory signaling, neutrophil chemoattraction, and leukocyte activation. Indeed, IL-36 has been implicated in the activation of the innate immune response [9, 11, 17].

Collectively, these findings indicate a potential role for IL-36 in the pathophysiology of CAD.

In this context, Tian et al [18] conducted an animal study in rats fed an atherogenic diet, in which atherosclerosis developed and atheromas were subsequently evaluated. The authors demonstrated that IL-36 stimulated inflammasome activation through the NLRP3 complex, caspase-1, and IL-1β, and that IL-36Ra suppressed this response, indicating a link between IL-36 and atherosclerosis.

Moreover, in an animal study, Zhang et al [19] demonstrated that atheromatous plaques showed increased IL-36Ra levels and that IL-36 was associated with a greater atheroma volume and greater macrophage content.

From a pathophysiological perspective, IL-1, IL-6, IL-10, ICAM-1, VCAM-1, and TNF, which may be produced in response to IL-36 stimulation, are recognized mediators involved in the development of coronary atheroma. The role of the innate immune response activated by IL-36 family cytokines in the context of CAD is poorly understood [3, 20, 21].

In our study of patients with coronary atheroma, circulating IL-36β and IL-36γ levels were higher, indicating increased production of these ILs and their detectability in peripheral blood. Thus, we demonstrated an association between CAD and elevated peripheral IL-36 levels.

In living humans, there is currently no standardized method for directly quantifying IL-36 levels within atheromas. However, elevated circulating levels in peripheral blood may serve as an indicator of atherosclerotic risk and warrant further investigation.

Serum IL-36α and IL-36γ levels have been shown to be elevated in patients with type 2 diabetes mellitus, correlating with obesity and insulin resistance [22].

In the present study, most of the included patients did not have diseases described in the literature as being associated with elevated IL-36 levels. Thus, it may be hypothesized that atherosclerosis is associated with peripheral elevation of these ILs.

Abnormal IL-37 expression has been shown to be associated with atherosclerosis and other systemic inflammatory diseases, and there is also evidence suggesting that IL-37 suppresses inflammation through IL-18Rα signaling [23]. IL-37 influences reverse cholesterol transport, which may prevent foam cell formation, and has a role in the regulation of macrophages, lipids, and homeostasis during the inflammatory response, indicating a possible protective effect [24]. Treatment with recombinant IL-37 prevents atherosclerosis and improves cardiac function after acute myocardial infarction [25, 26]. In our study, no differences in circulating IL-37 levels were observed between patients with chronic CAD and healthy controls.

### Conclusions

In our study, circulating IL-36β and IL-36γ levels were higher in patients with chronic coronary syndrome than in healthy controls; however, further studies are needed to clarify the role of these ILs. Increased IL levels in peripheral blood may, in the future, serve as a marker of clinical event risk in patients with CAD.

The main limitations of this study include its single-center design, the absence of data on potential associations between serum IL levels and clinical outcomes, and the empirically determined sample size. The findings regarding the association between IL-36 and the severity of atherosclerosis should be confirmed in a study with a larger number of patients; the current study serves to generate hypotheses. We did not evaluate the potential influence of thoracic anthropometrics (for example: a concave-shaped anterior chest wall, including those with pectus excavatum or others) on inflammatory burden and CAD.

## Data Availability

The authors declare that data supporting the findings of this study are available within the article.
